# CF-PEEK vs. Titanium Dental Implants: Stress Distribution and Fatigue Performance in Variable Bone Qualities

**DOI:** 10.3390/biomimetics10090619

**Published:** 2025-09-14

**Authors:** Nurdan Polat Sağsöz, Fahri Murat, Sema Nur Sevinç Gül, Abdullah Tahir Şensoy, Irfan Kaymaz

**Affiliations:** 1 Faculty of Dentistry, Atatürk University, 25240 Erzurum, Turkey; nurdan.sagsoz@atauni.edu.tr (N.P.S.); sema.sevinc@atauni.edu.tr (S.N.S.G.); 2 Department of Mechanical Engineering, Faculty of Engineering and Architecture, Erzurum Technical University, 25050 Erzurum, Turkey; fahri.murat@erzurum.edu.tr (F.M.); irfan.kaymaz@erzurum.edu.tr (I.K.); 3 Faculty of Mechanical Engineering, Delft University of Technology, Mekelweg 2, 2628 CD Delft, The Netherlands; 4 Department of Biomedical Engineering, Faculty of Engineering and Natural Sciences, Samsun University, 55420 Samsun, Turkey

**Keywords:** dental implants, finite element analysis (FEA), fatigue analysis, CF-PEEK, bone density

## Abstract

This study aims to evaluate the biomechanical behavior of titanium and carbon fiber-reinforced polyetheretherketone (CF-PEEK) dental implants under varying bone densities and loading conditions using finite element analysis (FEA). A single-tooth mandibular molar implant system was modeled, comprising titanium or CF-PEEK abutment and fixture, and surrounding bone structures with four configurations: (I) fully cortical bone, (II) 2 mm cortical layer with trabecular bone, (III) 1 mm cortical with high-density trabecular bone, and (IV) 1 mm cortical with low-density trabecular bone. Vertical and oblique static loads of 100 N were applied to simulate masticatory forces. FEA results revealed that titanium implants exhibited higher von Mises stress values in the implant and abutment under oblique loading, exceeding 400 MPa, while CF-PEEK components showed reduced stress but significantly higher strain levels. Cortical and trabecular bone surrounding CF-PEEK implants received more uniform stress distribution, potentially minimizing stress shielding effects. However, fatigue life analyses indicated that CF-PEEK abutment and screw components were more susceptible to mechanical failure under oblique loads, particularly in low-density bone models. In conclusion, CF-PEEK implants offer a more physiological load transfer to bone and reduced stress shielding compared to titanium. However, their structural reliability under complex loading, especially in low-quality bone conditions, requires careful consideration. These findings support the potential use of CF-PEEK in select clinical scenarios but highlight the need for further material and design optimization.

## 1. Introduction

Tooth loss and edentulism are prevalent oral health problems that impact masticatory and speech functions. Advances in the field of dental implant science have led to the predominance of implant-supported restorations as the most prevalent treatment option for restoring oral function, stabilizing occlusion and enhancing esthetics [1,2]. Despite the documented success rates of over 94% for implant-supported restorations over an extended time period [3], mechanical complications and failures remain a significant concern [4,5].

A pivotal element in determining the success or failure of a dental implant is the manner and degree of stress transfer to the surrounding bone [6]. The transfer is contingent upon the type of loading, the bone-implant interface, the shape and characteristics of the implant surface, and the quality and quantity of the surrounding bone [7]. Bone density is directly related to bone strength [8]. Misch et al. categorized bone density into four groups from D1 (dense cortical bone) to D4 (thin trabecular bone). It was determined that D1 bone is ten times stronger than D4 bone [9]. The four different bone types examined in the study were carefully selected to exemplify the anatomical and structural variations that can be encountered clinically in the mandibular posterior region [10]. Utilizing these four models facilitates the evaluation of implant–bone interaction variability across diverse clinical scenarios, with the outcomes demonstrating clinical generalizability. In this context, it is widely accepted that poor bone quality is a significant obstacle to the osseointegration of implants and a common cause of implant failure [11].

For a considerable duration, titanium has been regarded as the ‘gold standard’ material in the field of dental implantology. Nevertheless, despite their extensive utilization, titanium implants are not without their drawbacks. Notably, titanium has been observed to induce allergic reactions or other immune system dysfunction in susceptible individuals. Furthermore, the presence of gray discoloration of the peri-implant mucosa has the potential to result in an undesirable esthetic outcome in these areas. In addition to the inherent biological and esthetic limitations of the material, the mechanical properties of titanium have been demonstrated to be associated with the occurrence of certain clinical complications [12].

The modulus of elasticity of titanium (~110 GPa) is significantly higher than that of surrounding bone tissue, with cortical bone around 13 GPa and trabecular bone nearly 1.3 GPa [13,14]. This substantial mismatch between the elastic modulus of implant materials and native bone tissue is a critical factor contributing to fatigue failure and subsequent bone loss in dental implants. One of the primary consequences of this mechanical incompatibility is the phenomenon known as stress shielding. Stress shielding arises when the load transfer between the implant and the surrounding bone is unbalanced, leading to insufficient mechanical stimulation of the bone. Over time, this can result in reduced bone density, peri-implant bone resorption, and eventual implant loosening. The stress shielding effect is particularly pronounced in metallic implants with high elastic moduli and can adversely impact the osseointegration process. Furthermore, the resulting load imbalance may cause excessive micromovements at the implant–bone interface, promoting fibrous tissue encapsulation [13], compromising the mechanical integrity of the interface, and hindering successful osseointegration [15].

In recent years, new implant materials have been developed to overcome these limitations. Polyetheretherketone (PEEK) has emerged as a promising alternative to traditional metallic dental implant materials due to its favorable mechanical properties and biocompatibility [16]. Therefore, recent efforts have focused on reinforcing PEEK with high-strength fibers and modifying its surface to enhance osseointegration [17]. A number of studies conducted on large animal models have indicated positive results with regard to the biocompatibility of PEEK implants. Nevertheless, the bone-to-implant contact ratio of pure PEEK is lower than that of traditional materials such as titanium and zirconia. Research in this area remains limited, with only partial osseointegration reported, particularly in canine tibias and other animal models. In order to address this issue, the use of hydroxyapatite (HA) coatings and surface modifications has been shown to enhance the biological performance of PEEK, by increasing bone volume and mineralization. However, the validity of these findings is limited by the insufficient in vivo studies, highlighting the need for more comprehensive animal models to evaluate clinical applicability [18,19].

One of the key advantages of PEEK lies in its elastic modulus (~3–4 GPa), which more closely approximates that of human cortical and trabecular bone compared to titanium (~110 GPa) [20]. This mechanical compatibility allows for a more physiological stress transfer between the implant and surrounding bone, potentially minimizing the risk of stress shielding, a phenomenon often associated with high-modulus materials that can lead to localized bone resorption and implant failure [21]. Finite element analysis studies have demonstrated that PEEK and its reinforced composites (e.g., carbon- or glass-fiber-reinforced PEEK) can provide higher stress transmission to peri-implant bone, encouraging mechanical stimulation and maintaining bone density [22]. Despite these advantages, the relatively low modulus of pure PEEK may limit its structural performance under high functional loads [19]. Therefore, recent efforts have focused on reinforcing CF-PEEK with high-strength fibers and modifying its surface to enhance osseointegration. These developments position CF-PEEK as a biologically and mechanically promising material, particularly for use in patients with metal hypersensitivity or in esthetically sensitive areas.

The extant literature on the biomechanical effects of CF-PEEK, a material which has only recently been developed, is inconclusive due to the limited number of studies analyzing its effects at varying bone densities. The present study aims to address this knowledge gap by providing a detailed comparative analysis of the biomechanical behavior of titanium and CF-PEEK dental implant materials when used with different bone qualities. This analysis is conducted using finite element analysis. The present analysis is concerned with the evaluation of the stress and strain distributions at the implant–bone interface, micromotion and fatigue life, and the analysis of the effects on implant success [23]. To address the limited evidence in this field, the study compares CF-PEEK and titanium implants in four bone qualities under vertical and oblique loading, considering dynamic mastication cycles and applying fatigue analysis within a biomimetic load transfer framework. In this context, the incorporation of a biomimetic perspective emphasizes the replication of physiological stress transfer, aiming to identify implant material–bone quality combinations that best support osseointegration, ensure long-term stability, and reduce mechanical complications.

## 2. Materials and Methods

This study involved the virtual modeling and biomechanical preparation of a single-tooth dental implant system using computer-aided design (CAD) and finite element analysis (FEA). A three-part structure including the implant, abutment, and crown was designed and positioned within a simplified mandibular bone block to evaluate component interactions under physiological loading.

### 2.1. Three-Dimensional Modeling Procedure

**Figure 1:**
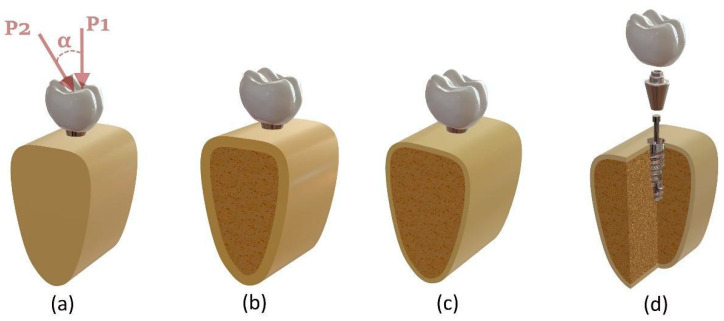
(**a**) Fully cortical bone (Model I) and loading directions: vertical (P1) and oblique (P2) at angle α; (**b**) 2 mm cortical bone (Model II); (**c**) 1 mm cortical with trabecular bone (Models III–IV); (**d**) exploded view of the implant system with crown, abutment, mini screw, and fixture.

A detailed 3D model of the dental implant system was created using SolidWorks 2024 (SolidWorks Corporation, Concord, MA, USA). As shown in Figure 1d, the model consists of four main components: (1) an anatomically shaped molar crown, (2) a tapered abutment, (3) a mini-screw, and (4) a threaded implant fixture. The implant fixture and abutment geometries were based on Nobel Active System implants (4.3 mm × 13 mm; Nobel Biocare AB) and multi-unit abutments with 0° (height: 3.5 mm) and 30° (height: 4.5 mm) angulations (Nobel Biocare AB), which were scanned using a 3Shape A3 scanner (BTech Innovation) and exported in STL format. These components were designed according to standard clinical dimensions used in restorations placed in the right mandibular first molar region.

The surrounding mandibular bone structure was designed in four different configurations to reflect realistic anatomical morphology. Model I consisted only of cortical bone, while Model II included 2 mm of cortical bone enclosing cancellous bone. Model III comprised a 1 mm-thick cortical layer with high-density cancellous bone (3507 MPa), and Model IV included 1 mm cortical bone with low-density cancellous bone (259 MPa). The first, second, and third configurations (note that Models III and IV share the same geometric structure) are shown in Figure 1a–c, respectively. The implant fixture was virtually inserted into the bone block and aligned perpendicular to the occlusal surface. Figure 1 illustrates the complete assembly of the implant system and its sectional representation within the bone block.

All components were exported as standard tessellation language (STL) files for subsequent analysis steps.

### 2.2. Boundary and Loading Conditions

All STL files were imported into ANSYS Workbench 2024 R2 (ANSYS Inc., Canonsburg, PA, USA) for meshing and finite element analysis. To simulate clinical masticatory loading, a vertical static force of 100 N (P1) was applied to the central fossa of the crown, representing a typical biting force [23]. Additionally, an oblique load of 100 N (P2) was applied at a 60° angle from the buccal to the lingual direction (α = 30°) to simulate off-axis loading conditions [24]. The load vector was aligned along the implant’s long axis to induce compressive stress at the implant–bone interface. The cross-sectional surfaces of the bone segment were fully constrained in all degrees of freedom.

A bonded contact was defined between the implant and the surrounding bone to represent a fully osseointegrated state. Likewise, a bonded interface was assigned between the abutment and crown [25,26]. Additionally, frictional interfaces were assigned between the mini screw and abutment, between the abutment and implant, as well as mini screw and implant using the same coefficient of 0.2 [27].

**Figure 2:**
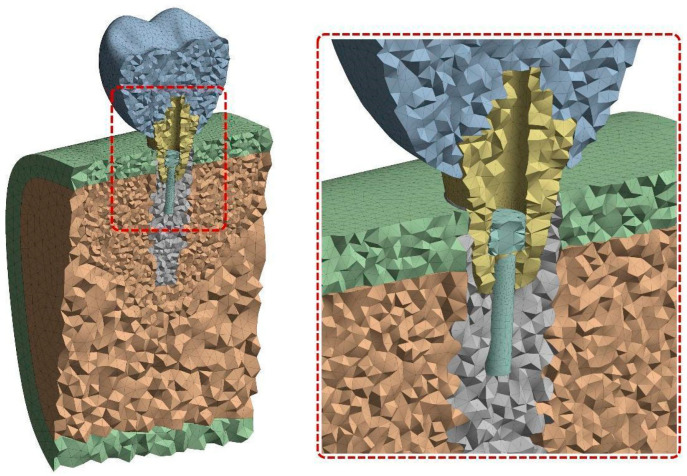
Cross-sectional view of the meshed implant system demonstrating element distribution across the crown, abutment, mini screw, implant, and bone.

First-order tetrahedral elements were used for mesh generation. The mesh sizes were defined as 0.5 mm for the implant and screw threads, 0.1 mm for the abutment and crown, and 1 mm for the bone structures, yielding a global average element size of approximately 1.4 mm. The mesh density was refined particularly in the implant–bone interface and prosthetic connection regions to enhance resolution. The final mesh contained approximately 570,000 nodes, with a total element count ranging between 350,000 and 400,000 depending on the configuration. In all mesh structures, the average element quality exceeded 0.81, and skewness values remained below 0.25. A cross-sectional view of the meshed model is presented in Figure 2.

The crown was modeled using feldspathic porcelain, while the abutment and implant screw were defined as Grade 23 titanium (Ti-6Al-4V ELI). The bone structure was represented by two separate isotropic materials to simulate cortical and cancellous tissues. All materials were modeled as homogeneous, isotropic, and linearly elastic. Although CF-PEEK can exhibit orthotropic properties due to fiber orientation, isotropic properties were adopted in this study to ensure comparability across implant materials and to remain consistent with previously published finite element analyses of dental implants [8,23,25,26]. Material properties such as elastic modulus and Poisson’s ratio for each component are listed in Table 1.

### 2.3. Fatigue Analysis

The fatigue behavior of the implant material was evaluated using an S–N curve (Wöhler curve) based on the von Mises equivalent stress amplitude as the governing parameter, which represents the relationship between the applied stress amplitude and the number of cycles to failure, typically plotted on a logarithmic scale. Two key parameters were considered in the fatigue analysis: the number of cycles to failure, which reflects the material’s endurance under cyclic loading, and the alternating stress, defined as the difference between the maximum and minimum stresses. These parameters are crucial in determining whether the material operates within the high-cycle fatigue regime, which is essential for preventing long-term mechanical failure in clinical applications [28].

**Figure 3:**
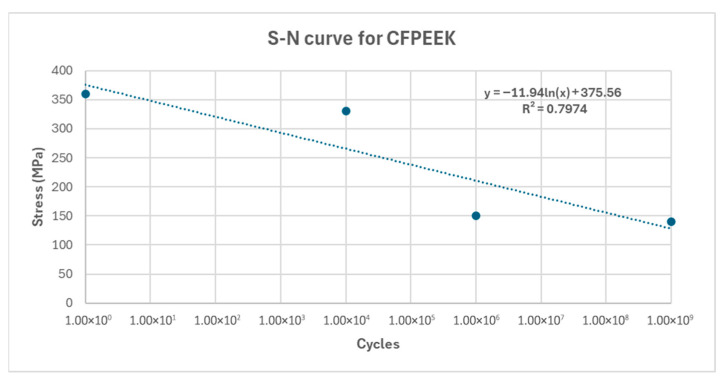
S–N curve of CF-PEEK showing experimental data (dots) and fitted logarithmic trend line (dotted line).

Various studies in the literature have investigated the fatigue behavior of CF-PEEK. For instance, Tai et al. (1995) examined the fatigue response of continuous fiber-reinforced AS-4/PEEK laminates, identifying different fatigue regimes depending on the applied stress ratio and load configuration [29]. Similarly, Li et al. (2021) provided experimental S–N data for short CF-PEEK using thermomechanical coupled fatigue tests [30]. However, in the present study, the fatigue data given Figure 3 were adopted from Martínez-Mondragón et al. (2024) [31].

Although various theories such as Goodman, Soderberg, and others have been suggested in the literature to account for the effect of mean stress in fatigue analysis, Tsai et al. [28] emphasized that the Gerber theory provided the best agreement with experimental results in dental implant systems. Therefore, in the present study, the Gerber approach was adopted to account for the influence of mean stress in the fatigue analysis. In addition, a load ratio (R = σmin/σmax) of 0.1 was implemented to simulate clinically relevant loading conditions.

## 3. Results

### 3.1. The Von Mises Stress Analysis Results

**Figure 4:**
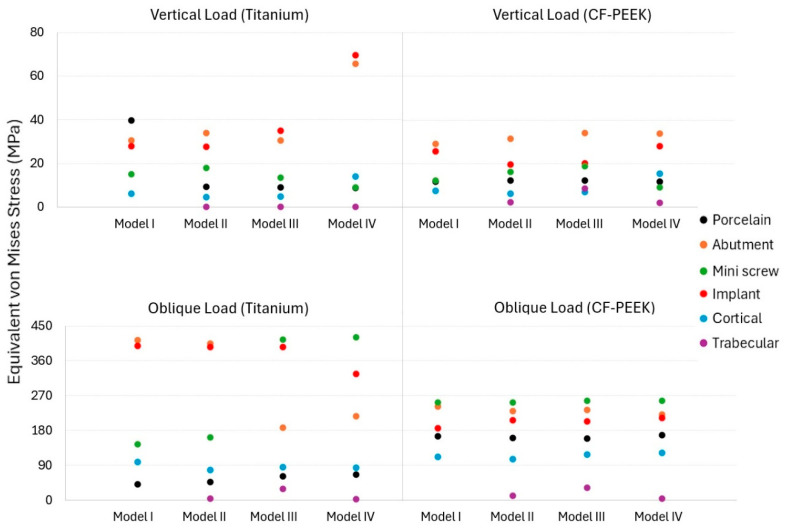
Equivalent von Mises stress results obtained from the FEA under different loading and material conditions across all components.

The von Mises stress distribution varied significantly between different components depending on the implant material, loading direction, and bone quality scenario, as shown in Figure 4. Under vertical loading, stress values remained low in the titanium system with porcelain crowns, except for Model I, while slightly higher values were observed in the CF-PEEK systems. This is likely due to increased deformation at the abutment-crown interface.

Both titanium and CF-PEEK groups showed the highest stress values among all prosthetic elements for abutment and implant components. In particular, titanium abutments under vertical loading exhibited stress values around 30–34 MPa (except for Model IV, which showed 65.6 MPa in the abutment), while CF-PEEK abutments exhibited similar or slightly lower values (28–31 MPa).

Higher stress levels (27–35 MPa) were observed in titanium implants under vertical loading (69.6 MPa in Model IV only), while lower stresses (19–25 MPa) were observed in CF-PEEK implants, which resulted in better load distribution due to the polymeric structure. Under oblique loading, stress values increased significantly, particularly in titanium implant systems. In titanium abutments and implants, excessive stress values exceeding 390 MPa were observed, particularly in Models I and II. In contrast, CF-PEEK implants and abutments exhibited significantly lower stress values (between 184 and 240 MPa).

Under vertical loads, titanium and CF-PEEK mini screws showed similar low stress values. Under oblique loads, titanium mini screws showed stress values of up to 420 MPa, while CF-PEEK showed values of 255 MPa.

Under oblique loading in the CF-PEEK groups, cortical bone was generally subjected to higher stress (up to 120 MPa), indicating that the load was transferred to the surrounding bone rather than concentrated within the implant components. Titanium implants have shown higher resistance to stress accumulation despite higher stress values that could contribute to localized bone resorption (stress shielding).

**Figure 5:**
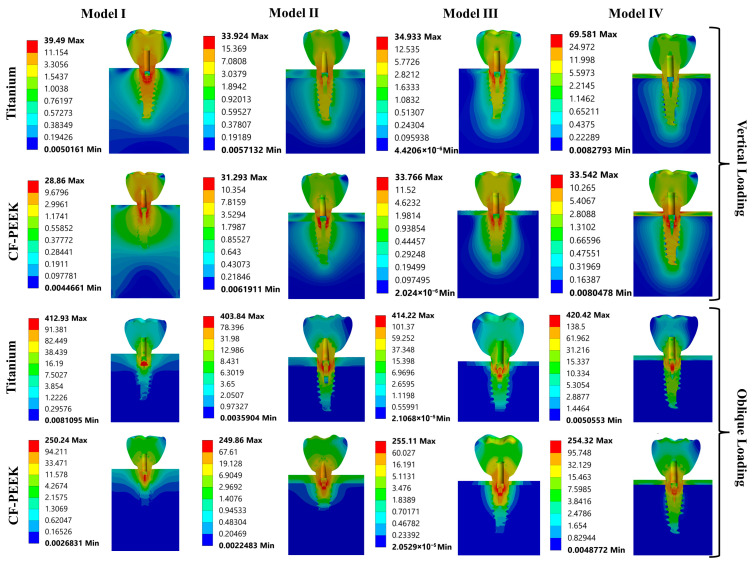
von Mises stress distributions obtained under vertical and oblique loading conditions for Titanium and CF-PEEK implant systems across four different bone density models.

As shown in Figure 5, in Model I, under vertical load, the highest stress value was observed in the porcelain of the titanium implant system and in the abutment of the CF-PEEK system. Under oblique load, the highest stress value was observed in the abutment of the titanium implant system and in the mini screw of the CF-PEEK implant system.

In Model II, under vertical load, the highest stress value was observed in the abutment of both systems. Under oblique load, the highest stress value was observed in the abutment of the titanium implant system and in the mini screw of the CF-PEEK implant system.

In Model III, under vertical load, the highest stress value was observed in the implant of the titanium implant system and in the abutment of the CF-PEEK system. Under oblique load, the highest stress value was observed in the mini screw in both systems.

In Model IV, under vertical load, the highest stress value was observed in the implant of the titanium implant system and in the abutment of the CF-PEEK system. Under oblique load, the highest stress value was observed in the mini screw in both systems.

The stress shielding value was calculated by taking the ratio of the maximum von Mises stress differences between the cortical bone and the implant to the maximum stress in the cortical bone. Significantly lower ratios were found in CF-PEEK implant systems compared to titanium. For example, in Model II under oblique loading, the ratio was 98.05% in CF-PEEK implant systems, while it rose to 406.41% in titanium systems. Under vertical loading, the ratios reached up to 750% in titanium systems (Model III), while in CF-PEEK systems, this ratio remained around 250%.

### 3.2. Strain Results

Strain distribution analysis shows differences between titanium and CF-PEEK implant systems in Table 2. Under vertical loading, titanium components exhibited lower strain values in all models, reflecting the material’s higher stiffness. For example, abutments in titanium systems exhibited strain levels below 250 µε, independent of cortical bone thickness. In contrast, CF-PEEK abutments generally exhibited higher strain magnitudes exceeding 1300 µε. Similarly, strain in CF-PEEK implants was consistently higher than in titanium implants, especially under vertical loading, reaching values approaching 1000 µε compared to approximately 300 µε for titanium.

Under oblique loading, the strain differences between materials have become more pronounced. Despite exhibiting lower stress values, CF-PEEK systems have experienced a significant increase in strain in almost all components. For example, the abutment strain value exceeded 10,000 µε in CF-PEEK in Model I, while it approached 2400 µε in titanium. The implant strain value also followed a similar trend, with CF-PEEK values in Model II exceeding 6900 µε under the same loading, nearly three times the titanium strain values.

Stress accumulation in peri-implant bone structures also exhibited material-dependent behavior. In CF-PEEK systems, both cortical and trabecular bone showed slightly higher stresses than titanium cases. In particular, in Model III, trabecular strain values in CF-PEEK systems reached up to 9450 µε. However, excessive strain was observed in the CF-PEEK mini screw, which showed strain levels around 14,000 µε under oblique loading.

### 3.3. Fatigue FEA Results

The fatigue analyses of the four models considered in this study were performed using a finite element-based approach, incorporating the S–N curves and fatigue limit values of Titanium and CF-PEEK. Two distinct loading conditions, vertical and oblique, were applied to evaluate the fatigue performance of each model. The fatigue damage values, which will be reported in the following sections, were obtained using Miner’s cumulative damage rule, where a value of 1.0 represents the attainment of the fatigue limit without failure. The equivalent cyclic stress was determined according to the von Mises criterion, as the loading conditions involved not only vertical compression but also oblique loading, introducing bending and combined stress states. Therefore, the use of the von Mises equivalent stress is essential to accurately account for the loading and its influence on fatigue behavior under simulated physiological chewing conditions. The results obtained under both loading scenarios are presented below.

#### 3.3.1. Vertical Loading Conditions

Under vertical loading conditions, the fatigue life for both Titanium and CF-PEEK models was calculated to be $1\times 10^{9}$ cycles. Additionally, the predicted damage value for all components in both material models was found to be 1.0, indicating that the fatigue limit was reached without any failure during the simulation.

**Figure 6:**
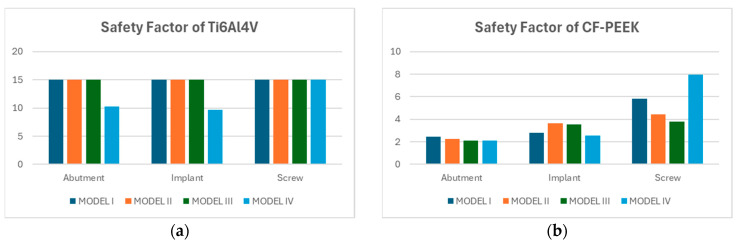
Safety factors for the models made from Titanium (**a**) and CF-PEEK (**b**) under vertical loading conditions.

Figure 6a presents the safety factors calculated for the abutment, implant, and screw components made of Titanium alloy across four model configurations. For Models I, II, and III, all components exhibited consistently high safety factors, reaching the upper threshold of 15. However, in Model IV, a noticeable decrease in safety factor was observed, particularly in the abutment and implant, which showed values of approximately 10.1 and 9.3, respectively. Despite this reduction, all safety factors remained well above the failure threshold, suggesting that Titanium maintains robust mechanical performance even under the altered conditions of Model IV. These findings reaffirm the alloy’s suitability for high-load dental applications.

Figure 6b illustrates the safety factors calculated for the abutment, implant, and screw components of the CF-PEEK dental implant system across four different models (Model I–IV). Among the three components, the screw exhibited the highest safety factors overall, with Model IV reaching a maximum value of 8.0, followed by 5.7 in Model I, 4.2 in Model II, and 3.5 in Model III. The implant component demonstrated moderately high safety factors, ranging from 2.4 to 3.2 across all models, with Model II showing the highest value. In contrast, the abutment showed consistently lower safety factors, with all four models yielding values close to 2.0.

#### 3.3.2. Oblique Loading Conditions

**Figure 7:**
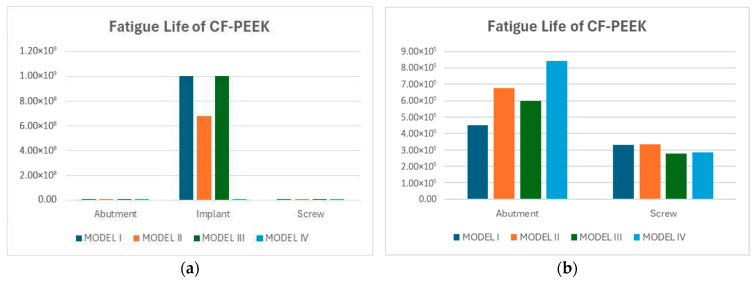
Fatigue lives of CF-PEEK models: (**a**) all components; (**b**) abutment and screw only, excluding implant to improve visual clarity.

Under oblique loading conditions defined by the ISO 14801 standard, the predicted fatigue life of the abutment, implant, and screw components was evaluated for both Titanium and CF-PEEK materials. Titanium exhibited an exceptionally high fatigue life across all components and model configurations, consistently reaching be $1\times 10^{9}$ cycles. In contrast, the fatigue life of the CF-PEEK components varied considerably across different models and component types. The implant consistently exhibited the highest fatigue life among all components, reaching up to 1.0 × 10^9^ cycles in Models I and III, except the implant having around $6\times 10^{8}$ cycles of fatigue life. In contrast, both the abutment and screw showed significantly lower fatigue lives across all configurations. To better visualize these differences, the implant results were excluded from a separate plot focused solely on the abutment and screw. In this view, the abutment demonstrated the most favorable fatigue life in Model IV (~8.5 × 10^5^ cycles), followed by Model II, while Model I yielded the lowest value (~4.6 × 10^5^). The screw consistently exhibited the shortest fatigue life among all components, with values ranging between 3.0 × 10^5^ and 3.9 × 10^5^ cycles across all models (Figure 7).

Fatigue damage values varied considerably between Titanium and CF-PEEK components across all model configurations. For Titanium, the damage values remained constant at approximately 1.0 for all components and models, indicating that the fatigue limit was reached without having any damage.

**Figure 8:**
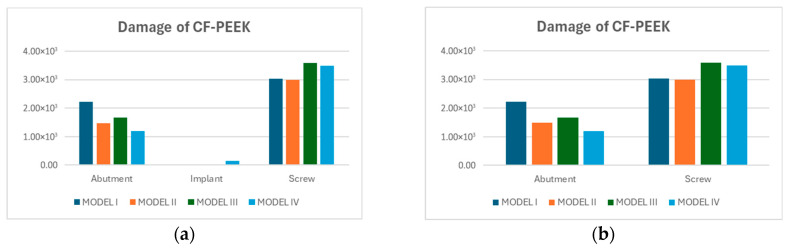
Damage values of CF-PEEK models: (**a**) all components; (**b**) abutment and screw only, excluding implant to improve visual clarity.

In contrast, CF-PEEK components exhibited substantially higher numerical damage values, particularly in the screw and abutment regions. While the implant consistently exhibited very low damage values typically below $5\times 10^{2}$ the abutment and screw regions showed significantly higher damage levels, exceeding $2\times 10^{2}$, as shown in Figure 8a. To enhance visual clarity and facilitate comparison, a separate figure given in Figure 8b was generated by removing the implant data. In this refined view, the screw emerged as the most critical component, with damage values reaching up to $3.8\times 10^{3}$ in Model III. The abutment followed, showing its lowest damage in Model IV ($1.3\times 10^{2})$ and the highest in Model 1 ($2.4\times 10^{3})$.

**Figure 9:**
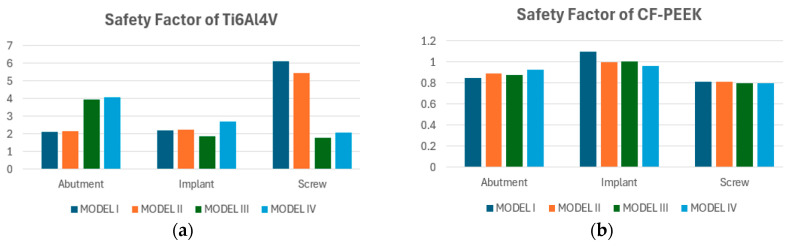
Safety factors for the models made from Titanium (**a**) and CF-PEEK (**b**) under oblique loading conditions.

The safety factor analysis demonstrated clear distinctions in fatigue performance between Titanium and CF-PEEK components across the four modeled configurations under oblique loading. Titanium consistently exhibited higher safety margins across all components. Notably, the screw in Model I reached a safety factor of approximately 6.3, while the implant and abutment, maintained values in the range of 2.1 to 4.1, depending on the model. Model III showed the highest safety factor for the abutment (~4.0), indicating favorable stress distribution. In contrast, CF-PEEK components operated with safety factors close to the critical design threshold of 1.0, particularly in the screw and abutment, which averaged between 0.85 and 0.95 across all models. The implant exhibited slightly higher values, with a peak of 1.15 in Model I, yet still close to the limit of allowable stress (Figure 9).

**Figure 10:**
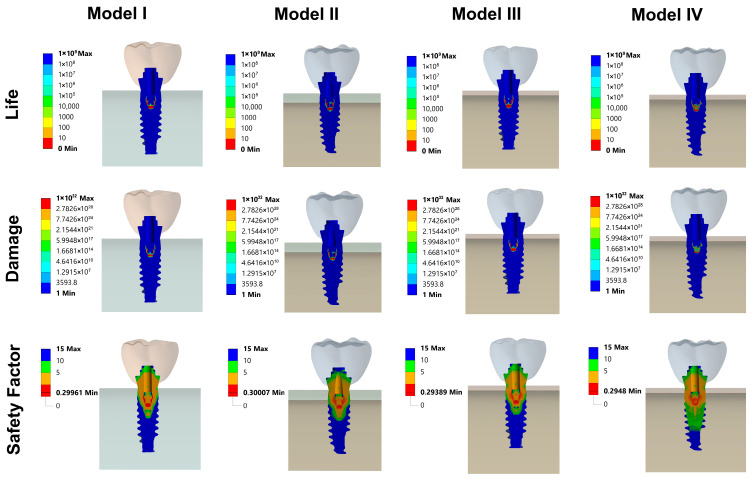
Fatigue FEA results. Life, damage and safety factor distribution on implant, abutment and mini screw section.

As seen in Figure 10, the fatigue results illustrate the distribution of life expectancy, damage, and safety factors across the implant, abutment, and mini screw components of all models. These findings highlight critical regions that are more susceptible to mechanical fatigue failure under cyclic loading conditions, providing valuable insights for improving the design and durability of the implant system.

## 4. Discussion

This study evaluated titanium and CF-PEEK dental implants in terms of stress and strain distribution under vertical and oblique loading across different bone qualities and fatigue analyses were conducted. Titanium implants showed higher stress concentrations, especially under oblique loading, which may occurred to stress shielding and localized bone resorption. CF-PEEK implants, with their lower elastic modulus, exhibited better load distribution and reduced stress peaks in bone, potentially promoting bone remodeling. However, both materials showed significantly higher strain levels in implant components, particularly when subjected to oblique loading. This raises concerns about mechanical stability and fatigue failure.

The findings indicated that the implant material exerts a substantial influence on the stress distribution within the implant and the surrounding bone tissue. The selection of material was found to be of particular criticality in low-density bone.

PEEK (polyetheretherketone) is a material of particular significance in the field of dental implantology due to its mechanical strength, biocompatibility, and radiolucent properties [32]. A critical advantage of the material is its modulus of elasticity, which is comparable to that of human bone. It is evident from the sources that PEEK’s modulus of elasticity is more closely aligned with that of cortical bone than with that of titanium [33,34]. This similarity has the potential to reduce the “stress shielding” phenomenon that has previously been observed in conventional titanium implants [35]. In this regard, Fabris et al. found that morse taper implants provided relatively lower bone stimulation, while PEEK implants caused excessive stress in the peri-implant region. In reinforced PEEK materials, stress distribution was found to be more balanced, and CF-PEEK monolithic implants were found to exhibit more suitable biomechanical behavior. Balanced stress levels reduce the risk of mechanical failure in implant systems and may induce bone remodeling in these areas [36]. Similarly, a study by Ouldyerou et al. found that titanium-PEEK (Ti-PEEK) implants are superior to traditional titanium implants in stress shielding and reducing bone resorption. Nevertheless, the mechanical strength of PEEK-based composite materials remains constrained, which is a substantial drawback with regard to long-term clinical success [35].

According to the data in the study, stress values in porcelain crowns remained low in vertical forces, while higher values were observed in CF-PEEK systems in oblique forces. While some studies suggest that different occlusal materials do not significantly affect stress distribution in implants, it has been noted that PEEK implant systems exhibit much higher deformation in their superstructures compared to titanium systems [37]. This situation may support increased deformation at the crown-abutment interface in CF-PEEK systems. It is also noted that the elastic modulus of crown materials may balance the high elastic modulus of the implant material.

In the present study, titanium abutments were subjected to high stresses under vertical and oblique loading, while CF-PEEK abutments showed similar or slightly lower values. However, it was observed that abutment strain under oblique loading was higher in CF-PEEK systems than in titanium. Similarly, it was noted that implant strain reached high values in CF-PEEK, with titanium values being nearly three times higher. These findings are consistent with some studies in the literature. For example, it has been reported that PEEK abutments and screws exhibit significantly higher strains than titanium [38]. Elsayed et al. (2025) also noted that polymeric implants (PEEK or PEKK-Polyetherketoneketone) exhibit lower von Mises stresses and higher total deformation values compared to titanium implants, attributing this to their lower elastic modulus [39]. The same study reported that polymer implants exhibit a more concentrated deformation pattern than titanium implants, particularly in the coronal regions. Yang et al. (2024) also reported that PEEK implants showed the highest deformation (57.35 μm), zirconium implants showed the lowest deformation (24.1 μm), and maximum deformation decreased as the elastic modulus of the implant increased [40]. However, another study noted that polymer (PEEK and PEKK-Polyetherketoneketone) prosthetic components exhibit higher deformation than titanium components, which is explained by their lower elastic modulus [39]. In the current study, the higher deformation of PEEK abutments is consistent with studies indicating that PEEK and PEEK components exhibit significantly higher strains than titanium. This situation shows that PEEK’s low elastic modulus causes greater deformation. Some studies indicate that PEEK abutments reduce stress in the abutment region by transferring stress to the implant and screw [22,41]. In the present study, it was observed that von Mises stress accumulated in the abutment under vertical loads and on the mini screw under oblique loads.

The study data show that titanium implant bodies have moderately higher stress levels, while CF-PEEK implants and abutments exhibited significantly lower stress values (between 184 and 240 MPa), confirming that the lower elastic modulus of CF-PEEK leads to reduced stress peaks under complex loading.

Some studies indicate that while PEEK implants themselves exhibit lower stress, stress values on the surrounding bone (particularly in terms of maximum shear stress (τmax) and minimum principal stress (σmin) may be slightly higher than those of titanium. In a related study, it was directly stated that a pure PEEK (PEEKp) implant showed a 68% reduction in von Mises stress compared to titanium and a 71% reduction compared to zirconia. Similarly, a 37% reduction was reported for carbon fiber-reinforced PEEK (PEEKc) and a 50% reduction for glass fiber-reinforced PEEK (PEEKg) [30]. This demonstrates that PEEK materials can significantly reduce the stress load on the implant itself. However, this does not necessarily mean that PEEK is “worse.” On the contrary, some authors suggest that this increased stress could actually provide a “balanced stress stimulus” for the bone and help prevent bone loss caused by stress shielding from highly rigid materials like titanium [42]. This indicates the potential to create a more biologically suitable loading environment at the implant–bone interface. However, there are conflicting findings in the literature on this subject. Some studies have indicated that PEEK implants do not offer an advantage over titanium implants in terms of stress distribution in the peri-implant bone, or even that 30% carbon fiber-reinforced PEEK implants produce higher stress values in cortical bone [43].

In line with the results of the current study, another study has reported that the von Mises stress values in the titanium subperiosteal implant system are approximately twice those in the 60% carbon fiber reinforced PEEK subperiosteal implant system. Under vertical loading, the titanium model exhibited a stress of 101.5 MPa, while the CF-PEEK model exhibited a stress of 57.0 MPa. It was also noted that the PEEK implant, which has a low Young’s modulus, exhibited the lowest values of maximum stresses in both the cortical bone and the implant. Similarly, it was observed that titanium implants exhibit approximately 20–30 MPa higher von Mises stress under vertical loading compared to polymeric implants [44]. These findings support our conclusion that titanium implants are subjected to higher stresses than CF-PEEK implants.

According to the results obtained, the von Mises stress values accumulated on the mini screw were found to be particularly high in CF-PEEK material under oblique loading, thereby increasing the strain values around the mini screw. The mini screw exhibiting strain at the 0.014 (14,000 microstrain) level under CF-PEEK and oblique loading may increase the risk of fatigue-related fracture. It has been reported that the highest stress concentrations in the implant and abutment are observed at the location where the abutment screw is seated [45]. In general, screw fractures are common, but implant body fractures are rarer. Titanium screws are reported to have higher fracture resistance than PEEK and 30% CF-PEEK screws in the literature [46]. Recent studies have demonstrated a close relationship between screw loosening and bone remodeling in the peri-implant region after prosthetic loading [47]. As demonstrated by Assoratgoon et al. [48], the remodeling process has the capacity to alter load transfer at the implant-abutment interface. This, in turn, has the potential to reduce screw preload and result in micromotion. In this context, the elevated strain values recorded during oblique loading, particularly in the CF-PEEK implant systems and low-density bone models examined in our study, although remaining within physiological limits, have the potential to adversely affect screw stability over time by influencing bone tissue remodeling. This finding demonstrates that, in addition to implant material properties, the quality and remodeling capacity of the surrounding bone should also be considered for prosthetic stability.

CF-PEEK implants have demonstrated a more favorable stress distribution in the peri-implant region, particularly in trabecular bone, due to their elastic compliance [49]. However, increased stresses observed in prosthetic components under oblique loading, particularly in CF-PEEK cases, have raised concerns about the long-term mechanical stability and fatigue performance of these polymer-based systems. The study results show that both cortical and trabecular bone in CF-PEEK systems exhibit slightly higher strains compared to titanium cases, but these values remain within physiologically tolerable limits. It was noted that PEEK implants exhibited significantly higher maximum strains in cortical bone, while titanium implants exhibited lower values [39]. This suggests that PEEK implants may more effectively support bone growth and adaptation.

In low-density bone scenarios, trabecular strain values reaching up to 9000 µε in CF-PEEK systems suggest increased mechanical stimulation. Bone tissue remains in a state of adaptation or “lazy zone” within a specific strain range (100–2000 µε), where bone apposition and resorption are in equilibrium [49]. The 1500–3000 µε range indicates mild overloading and bone growth, while values above 3000 µε indicate pathological overloading and the possibility of microfractures [50]. The 9000 µε value in our data is well above the pathological overloading threshold reported in the literature. In PEEK models, maximum strain values exceeding 3000 µε in cancellous bone have been accepted as the lower limit of physiological bone tissue absorption strain, and it has been noted that this situation may lead to excessive displacement of the implant and strain in the bone. This raises the risk that, especially in low-quality bone, these high strains may cause damage rather than benefit the bone.

The inclusion of cases exhibiting varying degrees of bone density is a crucial aspect of the present study. The present study determined that these differences may have different consequences for Ti and PEEK implant materials. CF-PEEK materials have demonstrated the potential to reduce the stress shielding effect by providing a more physiological and predictable stress distribution, particularly in lower-density bones such as D3 and D4. This is attributed to their elastic modulus, which is close to that of human bone, and their high mechanical strength. This demonstrates the ability of CF-PEEK to provide a more uniform stress distribution with the surrounding bone tissue, even under high masticatory forces, thanks to its elastic modulus, which is close to that of bone. The findings of the present study are also supported by the results of similar studies in the literature regarding the stress shielding potential and biocompatibility of CF-PEEK [51]. Additionally, the lower stiffness and higher flexibility of CF-PEEK compared to titanium are hypothesized to be advantageous in terms of the implant’s positive effects on bone remodeling in the long term.

This study employed a finite element-based fatigue analysis to evaluate the mechanical behavior of dental implant assemblies made from Titanium and CF-PEEK under vertical and oblique loading conditions. The simulations incorporated material-specific S–N curves and fatigue limit values in accordance with ISO 14801, which requires implant systems to endure at least 5 million cycles under simulated intraoral loading. The results revealed clear differences in fatigue performance and safety margins between the two materials, with implications for their clinical viability.

Under vertical loading, both Titanium and CF-PEEK components achieved fatigue lives of $1\times 10^{9}$ cycles, comfortably exceeding the ISO 14801 threshold. Corresponding fatigue damage values remained at 1.0 for all components, indicating that while the fatigue limit was reached, no failure occurred. Titanium demonstrated exceptional structural reliability, as reflected by safety factors that reached the upper limit of 15 in Models I through III, and only slightly decreased in Model IV due to altered bone support conditions. CF-PEEK, although operating with lower safety margins, also remained within acceptable thresholds under vertical loading. Safety factors for CF-PEEK implants ranged from 2.0 to 8.0, with the screw component in Model IV showing the highest value. These results suggest that while Titanium offers substantial fatigue behavior, CF-PEEK can perform adequately under fatigue loads.

In contrast, oblique loading, which more closely replicates the off-axis forces experienced during mastication, revealed a significant divergence in fatigue performance. Titanium components consistently reached $1\times 10^{9}$ cycles across all models, with fatigue damage values remaining constant and safety factors ranging from 2.1 to 6.3. This consistent behavior confirms the dental implant system made of Titanium robustness even under complex loading scenarios. Conversely, CF-PEEK components, particularly the abutment and screw, exhibited lower fatigue lives under the same conditions. Several models, including Model I and III, yielded fatigue lives for the screw component in the range of $3\times 10^{5}$ to $4\times 10^{5}$ cycles, falling well below the ISO 14801 minimum. The abutment performed slightly better, with Model IV reaching up to $8.5\times 10^{5}$ cycles, but still short of the required 5 million cycles. Only the implant component in Model I and Model III approached or exceeded $1\times 10^{9}$ cycles under oblique loading conditions.

Fatigue damage values for CF-PEEK under oblique loading further underscored these concerns. The screw and abutment exhibited damage levels exceeding $2\times 10^{3}$, indicating significant accumulation of cyclic strain energy. In contrast, the implant consistently exhibited damage below $5\times 10^{2}$, confirming its relatively stable performance. To better illustrate the variation among components, implant results were excluded in a separate plot, which clearly revealed the screw as the most vulnerable component in the CF-PEEK assembly.

The safety factor analysis further reinforced these findings. For titanium, safety factors under oblique loading remained well above the critical threshold across all components and models, confirming its robust fatigue performance. However, for CF-PEEK, safety factors dropped significantly and approached critical levels. The abutment and screw showed values consistently between 0.85 and 0.95, suggesting that these components were operating close to their allowable fatigue limits. The implant component demonstrated marginally improved performance, reaching a maximum safety factor of 1.15 in Model I; however, it still exhibited a limited safety margin under oblique loading conditions. These safety margins indicate that while CF-PEEK may not fail immediately under oblique loading, its design tolerance is narrow, making it sensitive to variability in load, geometry, or bone support.

Fatigue analysis under vertical loading demonstrated that both Ti-6Al-4V and CF-PEEK implants reached a fatigue life of 1 × 10^9^ cycles, corresponding to approximately 1520 years, with a predicted damage value of 1.0, indicating that the fatigue limit was attained without failure. Under oblique loading according to ISO 14801, titanium components maintained the same exceptionally high durability above 10^9^ cycles, while CF-PEEK implants exhibited slightly reduced but still clinically sufficient lifetimes of about 6 × 10^8^–10^9^ cycles, equivalent to 914–1520 years. By comparison, the CF-PEEK abutment and screw showed markedly shorter fatigue lives, ranging from about 3.0 × 10^5^ to 8.5 × 10^5^ cycles, which corresponds to 0.45–1.3 years, thereby highlighting these components as potential critical limitations in long-term performance [52,53].

An important insight was gained from the comparison of Model III and Model IV, which were geometrically identical but differed in trabecular bone stiffness. Model III, which used a higher elastic modulus for trabecular bone, yielded higher safety factors and extended fatigue lives, especially in CF-PEEK components. This suggests that the mechanical properties of peri-implant bone critically affect load distribution and damage accumulation within the implant system. The reduced stiffness of trabecular bone in Model IV resulted in higher load transfer and localized stress concentrations within the implant structure, which is especially detrimental for lower-strength materials like CF-PEEK.

The present study is subject to certain limitations, including the assumptions inherent to FEA. FEA studies require more accurate modeling of material properties (anisotropy, viscoelasticity, non-homogeneity) and the realistic structure of bone. The biological response of bone tissue to mechanical loads is dependent upon the direction and type of stress applied. As evidenced by extant literature, physiological levels of tensile and compressive stresses have been demonstrated to facilitate bone formation and adaptive remodeling processes by stimulating osteoblast activity. However, it is noteworthy that shear stresses which exceed physiological limits may exert deleterious effects on the bone-implant interface. Another limitation of this study is the lack of direct evaluation of corticalization. While corticalization may initially enhance implant stability under functional loading, excessive corticalization has been linked to reduced bone remodeling capacity and marginal bone loss [54]. Given that implant material and elastic modulus play a role in this process, further clinical studies are required to clarify whether PEEK implants can provide a more physiological balance compared to titanium. Additionally, long-term in vitro and in vivo studies are recommended to clinically validate the results obtained. Furthermore, the evaluation was confined to specific implant geometries, and the geometry of the implant and the entire system limits the generalizability of the findings. This finding underscores the necessity for clinical validation of the simulations. Nevertheless, the long-term biomechanical durability and biological response of materials such as CF-PEEK remain insufficiently elucidated.

Another limitation of the present study is that no surface treatment was incorporated into the implant models. Since surface modifications are known to enhance osseointegration, improve biocompatibility, and reduce the modulus of elasticity, the present findings should be interpreted within the scope of untreated implant surfaces and material-only comparisons. Other important aspect concerns the potential effect of sliding at implant- abutment-miniscrew interfaces when relatively low coefficients of friction are assumed. Even limited sliding can lead to micromotion, which may accelerate fretting fatigue, induce localized shear stresses, and promote progressive damage at the abutment–screw junction. Such shear-induced mechanisms are considered key contributors to abutment screw loosening and fracture in clinical practice. While our finite element model primarily assessed stress, strain, and fatigue safety factors, future studies should incorporate advanced contact formulations and experimental testing to better capture the influence of sliding-induced shear damage on the long-term durability of implant systems.

Consequently, while the bone-compatible deformation behavior and stress shielding potential of CF-PEEK implants offer significant advantages, careful consideration should be given to their use under high-load conditions due to the high strains in critical prosthetic elements such as abutments and screws. The consequences of these elevated strains on potential micromotion and fatigue fractures, particularly in regions exhibiting inadequate bone quality, are significant factors to be taken into account in the material’s clinical utilization.

In summary, the findings reaffirm Titanium’s position as the gold standard for dental implant applications, offering high fatigue resistance and safety across a wide range of loading and anatomical conditions. Although CF-PEEK demonstrates biomechanical advantages, including a bone-matching elastic modulus that may reduce stress shielding, its fatigue life and safety factor under oblique loading fall below clinically acceptable thresholds, particularly for abutment and screw components. To realize CF-PEEK’s full potential in dental applications, future work should focus on material enhancement (e.g., increased fiber volume, oriented reinforcements), design optimization, and customized geometries that mitigate critical stress regions and extend fatigue life beyond the ISO 14801 limit.

## 5. Conclusions

This study demonstrates that implant material and bone quality significantly influence the biomechanical performance of dental implants under functional loading. Titanium implants showed superior structural stability and fatigue resistance, particularly under oblique loading, but also exhibited higher stress concentrations that may contribute to stress shielding. In contrast, CF-PEEK implants enabled more uniform stress distribution and better biomechanical compatibility with surrounding bone, especially in low-density conditions. However, their reduced safety factor and fatigue life in critical components such as the abutment and mini screw indicate a need for further design optimization. These findings suggest that CF-PEEK, while promising, must be carefully evaluated based on bone quality and loading conditions to ensure clinical success.

## Figures and Tables

**Table 1:** Mechanical properties used in the FEA model.

Material	Elasticity Modulus [GPa]	Poisson’s Ratios	Ref
Feldspathic porcelain	82.8	0.35	[8]
Implant	110	0.35	[8]
Abutment	110	0.35	[8]
Cortical Bone	13.7	0.3	[25]
Trabecular Bone (High Density)	3.507	0.3	[26]
Trabecular Bone (Low Density)	0.259	0.3	[26]
%30 CF-PEEK	18	0.39	[23]

**Table 2:** Microstrain (µε) results of the components under different loading conditions and material properties.

	**Model I**				**Model II**			
	Vertical		Oblique		Vertical		Oblique	
	Titanium	CF-PEEK	Titanium	CF-PEEK	Titanium	CF-PEEK	Titanium	CF-PEEK
Porcelain	232.42	62.407	424.89	1596.5	111.01	65.467	378.11	1529.2
Abutment	231.23	1320.8	2381.5	10,019	217.9	1384.5	2405.2	9709.8
Implant	195.05	995.79	3312.5	5380.2	217.21	823.95	3213.6	6929.7
Mini screw	71.237	355.39	1311.1	13,992	79.65	405.58	1477.9	13,943
Cortical	226.01	176.23	1966.7	2854	168.74	270.99	2029	3174.2
Trabecular					383.48	372.31	711.82	1061.5
	**Model III**				**Model IV**			
	Vertical		Oblique		Vertical		Oblique	
	Titanium	CF-PEEK	Titanium	CF-PEEK	Titanium	CF-PEEK	Titanium	CF-PEEK
Porcelain	45.18	60.80	461.33	1521.70	44.53	59.78	507.21	1608.90
Abutment	186.25	1587.10	1342.80	9848.50	401.09	1067.70	2075.90	9445.30
Implant	291.59	993.35	3207.20	5859.90	330.62	1386.40	2723.30	6299.10
Mini screw	49.24	439.86	3781.50	14,228.00	27.77	263.56	3815.70	14,187.00
Cortical	160.51	259.83	1700.30	2523.10	776.12	889.67	2146.00	3503.70
Trabecular	1211.10	2556.90	8702.20	9450.90	5329.30	4638.80	7824.70	6562.50

## Data Availability

The original contributions presented in the study are included in the article, further inquiries can be directed to the corresponding author.
